# Rapid dissemination of human T-lymphotropic virus type 1 during primary infection in transplant recipients

**DOI:** 10.1186/s12977-015-0236-7

**Published:** 2016-01-08

**Authors:** Lucy B. M. Cook, Anat Melamed, Maria Antonietta Demontis, Daniel J. Laydon, James M. Fox, Jennifer H. C. Tosswill, Declan de Freitas, Ashley D. Price, James F. Medcalf, Fabiola Martin, James M. Neuberger, Charles R. M. Bangham, Graham P. Taylor

**Affiliations:** Section of Virology, Department of Medicine, Imperial College London, Norfolk Place, London, W2 1PG UK; Department of Biology and Hull York Medical School, Centre for Immunology and Infection, University of York, York, UK; HIV and Antiviral Group, Public Health England, 61, Colindale Avenue, London, UK; Department of Renal Medicine, Manchester Royal Infirmary, Central Manchester NHS Foundation Trust, Oxford Road, Manchester, UK; Department of Infection and Tropical Medicine, Royal Victoria Infirmary, Newcastle-upon-Tyne, UK; John Walls Renal Unit, Leicester General Hospital, Gwendolen Road, Leicester, UK; Organ Donation and Transplantation, NHS Blood and Transplant, Bristol, UK

**Keywords:** HTLV-1, Proviral load, Clonality, 2LTR DNA circles, Organ transplantation, Raltegravir, Zidovudine

## Abstract

**Background:**

Human T-lymphotropic virus type 1 (HTLV-1) infects an estimated 10 million persons globally with transmission resulting in lifelong infection. Disease, linked to high proviral load, occurs in a minority. In established infection HTLV-1 replicates through infectious spread and clonal expansion of infected lymphocytes. Little is known about acute HTLV-1 infection. The kinetics of early HTLV-1 infection, following transplantation-acquired infection in three recipients from one HTLV-1 infected donor, is reported. The recipients were treated with two HTLV-1 enzyme inhibitors 3 weeks post exposure following the detection of HTLV-1 provirus at low level in each recipient. HTLV-1 infection was serially monitored by serology, quantification of proviral load and HTLV-1 2LTR DNA circles and by HTLV-1 unique integration site analysis.

**Results:**

HTLV-1 antibodies were first detected 16–39 days post-transplantation. HTLV-1 provirus was detected by PCR on day 16–23 and increased by 2–3 log by day 38–45 with a peak proviral doubling time of 1.4 days, after which steady state was reached. The rapid proviral load expansion was associated with high frequency of HTLV-1 2LTR DNA circles. The number of HTLV-1 unique integration sites was high compared with established HTLV-1 infection. Clonal expansion of infected cells was detected as early as day 37 with high initial oligoclonality index, consistent with early mitotic proliferation.

**Conclusions:**

In recipients infected through organ transplantation HTLV-1 disseminated rapidly despite early anti-HTLV-1 treatment. Proviral load set point was reached within 6 weeks. Seroconversion was not delayed. Unique integration site analysis and HTLV-1 2LTR DNA circles indicated early clonal expansion and high rate of infectious spread.

## Background

Human T-lymphotropic virus type-1 (HTLV-1) is associated with two main clinical disorders that arise in 5–8 % of carriers: HTLV-1 associated myelopathy (HAM), a progressive, inflammatory, spastic paraparesis, and adult T cell leukaemia/lymphoma (ATLL), an aggressive malignancy of CD4+ T-lymphocytes. The majority of infection occurs through sexual intercourse or from mother-to-child during breast feeding but infection from unscreened blood transfusions or organ transplants can occur [1]. Little is known about early HTLV-1 infection, which is asymptomatic, but data from recipients of infected blood transfusions suggest that most seroconversion occurs within 2 months [2]. Knowledge of the course of early HTLV-1 infection in immunosuppressed individuals is limited to case reports of HTLV-1-associated disease following infection at the time of organ transplantation with infection usually not recognised until the onset of symptoms [3–12].

Evidence from phylogenetic studies and integration site analysis reveals two routes by which HTLV-1 propagates within the host [13–16]: infectious spread, where the virus spreads from cell-to-cell through the formation of a virological synapse between infected and uninfected CD4+ T-cells [17], resulting in integration of the HTLV-1 provirus in a new genomic location in the newly infected host cell; and mitotic proliferation of infected CD4+ T-lymphocytes, which gives rise to clonal populations of infected CD4+ cells that can be identified and quantified by their unique genomic integration site [16].

The relative contributions of infectious and mitotic spread at different time points of infection are not known; this knowledge is required for designing rational treatment protocols. HTLV-1 viral RNA is rarely found in human plasma [21, 22], which is not infectious [23]. In HIV infection, viral episomes containing two long terminal repeats (2LTR DNA circles) are formed after completion of viral cDNA synthesis and translocation of the viral genome to the host cell nucleus, where recombination and direct ligation lead to the formation of episomes. For HIV there is evidence that these 2LTR DNA circles are a surrogate marker of ongoing viral replication [18–20] in the absence of detectable viral RNA. HTLV-1 2LTR DNA circles have not been studied previously.

Since late 2002, all blood donations in the UK have been screened for HTLV-1 [24] but real-time screening of organ donors only became universal in 2012, after the events reported here. We report the investigation and management of three transplant recipients exposed to HTLV-1 through solid organ transplantation from a single donor, which leads to new insights into the early spread of HTLV-1 infection in vivo.

## Results

### Clinical cases (Table 1)

The liver and both kidneys were retrieved from a deceased female Caucasian donor, who was not known to carry, and had no risk factors for, HTLV-1 infection. The organs were transplanted in accordance with UK Blood and Transplant service protocols to three HLA class-matched male recipients. At the time of organ retrieval the donor HTLV status was reported as ‘awaited’ but after transplantation the HTLV-1 seropositive status of the donor was detected and confirmed, following which the recipients were informed. No suitable samples for quantifying the proviral load of the donor were available.

**Table 1:** Clinical details of transplant recipients

	Case 1	Case 2	Case 3
Primary organ pathology	Alcoholic liver disease	Tubulo-interstitial nephritis with focal sclerosis	End stage renal failure of unknown aetiology (diabetes/hypertension)
Age at transplantation (years)	58	48	57
Ethnicity	Caucasian	Black Caribbean	Indian
Organ transplanted	Liver	Kidney	Kidney
Class 1 HLA type	A01, A24, B08, B15, C03, C07 DR1, DR3 DQ2, DQ5	A3, A34, B51, B71, Cw3, Cw16, DR13, DQ7	A3, A24; B52, B55; Cw1, Cw12; DR10 DR14; DQ5
Peri-operative immune suppression	Basiliximab Methylprednisolone	Basiliximab Methylprednisolone	Basiliximab Mycophenolate, Tacrolimus
Post operative immune suppression	Mycophenolate, tacrolimus	None	Tacrolimus, prednisolone
Day post transplant antiretrovirals commenced	Day 19	Day 17	Day 26
Dose of antiretrovirals	Zidovudine 250 mg bd Raltegravir 400 mg bd	Zidovudine 100 mg tds Raltegravir 400 mg bd	Zidovudine 100 mg tds Raltegravir 400 mg bd
Day antiretrovirals stopped	Day 66	Day 43	Day 80
Day organ removed	Not applicable	Day 0	Day 48
Indication for organ removal	Not applicable	Life-threatening intra-operative haemorrhage	Rejection/failure

b.d. *Bis in die* (twice a day), t.d.s. *ter die sumendum* (three times daily)

Clinical details of the recipients are summarised in Table 1. In one patient, the transplanted kidney was explanted within 12 h because of life-threatening haemorrhage (unconnected with the infection); the other kidney recipient developed allograft rejection and so underwent explantation. The liver recipient, treated with standard immunosuppression, remains well with normal graft function.

HTLV-1 infection was diagnosed by HTLV-1 DNA PCR in all three recipients who were then commenced on zidovudine and raltegravir, which inhibit HTLV-1 reverse transcriptase [25, 26] and integrase [27] respectively, with the aim of limiting early infectious spread. Antiretroviral treatment, given for 24–54 days, was tolerated well by all recipients who, at 30 months post-transplantation, have no evidence of HTLV-1-associated disease.

### HTLV-1 seroconversion (Fig. 1)

**Fig. 1:**
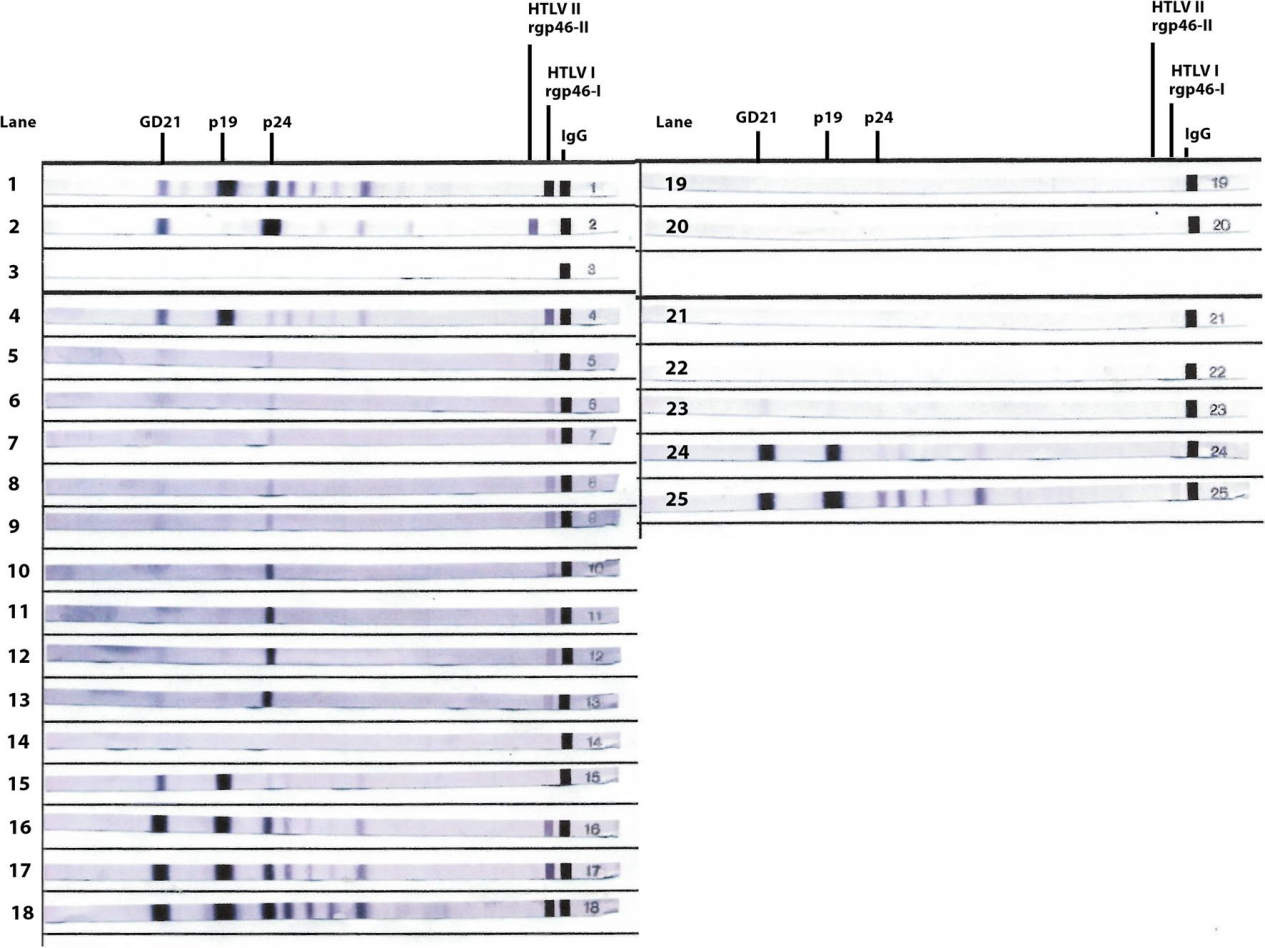
Western blots (Genelabs HTLV 2.4) of antibodies to natural and recombinant HTLV-1 antibodies. To assist with interpretation, only relevant HTLV-1/2 antigens have been highlighted. GD21 is a recombinant p21 transmembrane envelope protein; rgp46-1 and rgp46-2 are recombinant gp46 surface proteins specific for HTLV-1 and HTLV-2, respectively. p19 and p24 are group antigens (gag) from the nucleus. HTLV-1 positive control shown in *lanes 1* and *4*, HTLV-2 positive control in *lane 2*, negative control in *lane 3*. Case 1 (*lanes 5*–*13*) days 16, 32, 46, 71, 74, 186, 214, 242 and 270 post transplantation, shown in sequence. Case 2 (*lanes* *14*–*18*) days 16, 39, 95, 136 and 254 post transplantation, shown in sequence. Case 3 (*lanes* *19*–*25*) days.16, 23, 30, 37, 75, 145 and 208 post transplantation, shown in sequence

In case 1 the first detection of anti-HTLV-1 antibodies was 16 days post-transplantation. The enzyme linked immunoassay (EIA) sample/cut-off (S/CO) optical density was 6.88 and faint bands indicative of antibodies to p24 (gag), rgp46-1 (env) and anti-GD21 (env) were seen on the western blot (Fig. 1, lane 5). Anti-p19 (gag) was first detected at day 32 (Fig. 1, lane 6). In case 2 anti-HTLV-1 antibodies were first detected on day 39 (S/CO 13.5) at which time the western blot revealed a strong response to GD21 and p19 with faint anti-p24 (Fig. 1, lane 15). Anti-rgp46-1 was detected at day 95 (Fig. 1, lane16). In case 3 although anti-HTLV-1 antibodies were detected by EIA at Day 16 (S/CO 1.88) this could not be confirmed by western blotting until anti-GD21 was first faintly detected on day 75 (Fig. 1 lane 23). Strong responses to GD21, p19 and p24 were present by day 145 and a faint anti-rgp46-1 band at day 208 (Fig. 1 lanes 24, 25).

### HTLV-1 long terminal repeat (LTR) sequence identity

Alignment of 442 nucleotides of the HTLV-1 5′LTR showed 100 % sequence identity between the three individuals, consistent with a common viral source of infection.

### HTLV-1 proviral load and doubling time (Fig. 2)

At 16 days post-transplantation HTLV-1 provirus could not be detected by quantitative PCR (qPCR) in any of the recipients (<0.01 % PBMC infected). However, by nested PCR provirus was detectable and estimated at 0.003 % PBMC in Case 1 (liver transplant) and at 0.01 % in Case 2 (kidney transplant) but remained undetectable in Case 3 (kidney transplant) until day 23 when it became detectable at 0.01 % by qPCR. HTLV-1 proviral load increased in each case by 2–3 logs between days 16–23 and days 38–45, after which a steady state was reached with about 1 % PBMCs infected. Since it is known that there is a single copy of HTLV-1 integrated into each infected cell [28], the proviral load between the early time points can be used to estimate the doubling time of HTLV-1 infected CD4+ T-lymphocytes in the first month following infection which at its peak was a median of 1.43 days (range 1.1–2.9 days). The absolute lymphocyte count remained in the low normal range during this period (data not shown).

**Fig. 2:**
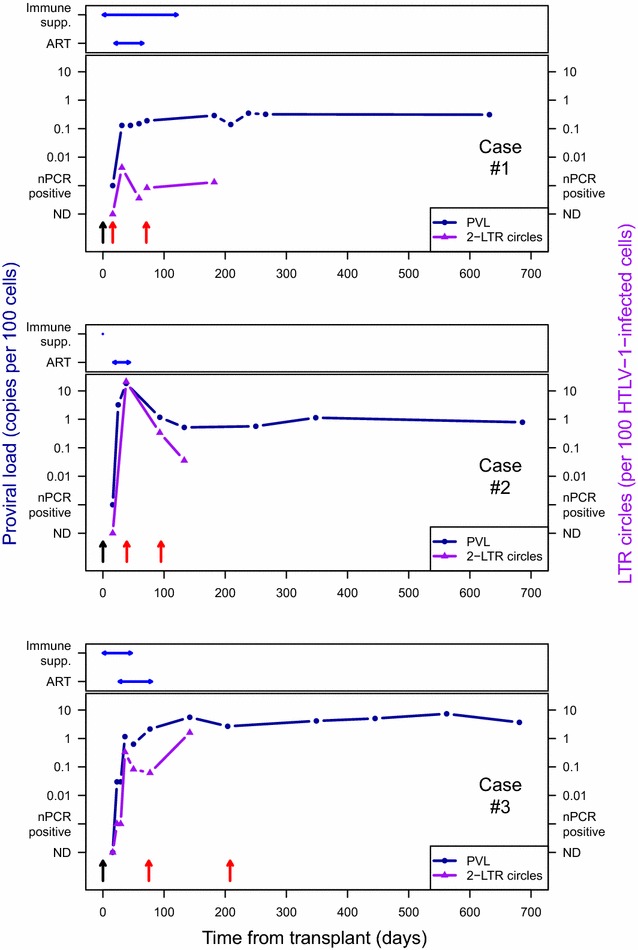
Time courses for case 1 (*upper panel*), case 2 (*middle panel*) and case 3 (*lower panel*) indicating the period of immunosuppressive (Immune Supp.) and antiretroviral therapy (ART) in relation to transplantation (*black arrow*), HTLV-1 seroconversion (*first* *red arrow*—first detection of anti-HTLV-1 Ab; *second red arrow*—all anti-HTLV-1 ab essential to confirm and type infection detected), HTLV-1 proviral load and frequency of HTLV-1 2LTR DNA circles per 100 HTLV-1 infected cells

### HTLV-1 2LTR DNA circles (Fig. 2)

In case 1: 2LTR DNA circles were measured at five time points (days 16, 31, 45, 72 and 182). They were undetectable at day 16, peaked at day 31 (4.2 × 10^−5^ 2LTR DNA circles/infected cell) and plateaued from day 45 (3.6 × 10^−6^ 2LTR DNA circles/infected cell).

In case 2: 2LTR DNA circles were measured at four time points (days 16, 38, 93 and 133). They were undetectable at day 16, peaked at day 38 (0.21 2LTR DNA circles/infected cell) and declined by the next time point at day 93 (3.3 × 10^−3^ 2LTR DNA circles/infected cell) and continued to decline (day 133, 3.6 × 10^−4^ 2LTR DNA circles/infected cell).

In case 3: 2LTR DNA circles were measured at seven time points (days 16, 23, 29, 36, 50, 77 and 142) and were undetectable at day 16, with an initial peak at day 36 (3.3 × 10^−3^ 2LTR DNA circles/infected cell) which had declined by next time point (day 50, 8.3 × 10^−4^ 2LTR DNA circles/infected cell) followed by a second peak at day 142 (0.16 2LTR DNA circles/infected cell) coinciding with a peak in the proviral load.

In all three cases, longitudinal analysis shows that the frequency of 2LTR DNA circles peaked between days 31 and 38 post-transplant at the same time as the initial peak in proviral load and whilst the patients were taking HTLV-1 reverse transcriptase and integrase inhibitors. The reduction in frequency of 2LTR DNA circles thereafter was greater than the reduction in proviral load (Fig. 2).

### Unique integration site (UIS) analysis (Fig. 3)

Each clone of infected cells can be defined by a unique genomic integration site [28] and the relative abundance, or proportion of the proviral load contributed by each clone, is represented by the size of the respective sector in a pie chart (Panel A). The five most abundant integration sites at the early time points are colour-coded and can be tracked over time with regard to absolute abundance (Panel B). At the earliest available time points HTLV-1 proviral load was too low to undertake high-throughput sequencing (HTS). HTS revealed that no integration site was found in more than one recipient, indicating that the observed integration sites were not persistent donor lymphocytes.

**Fig. 3:**
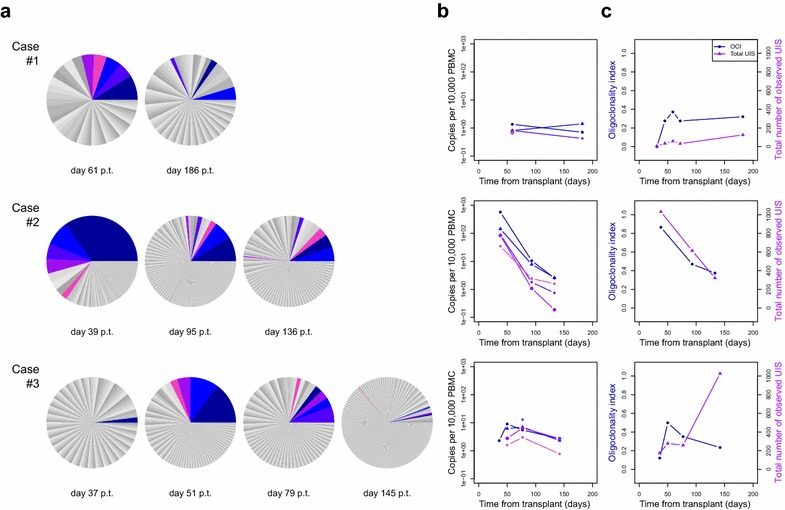
**a** shows the relative abundance of unique integration sites in each case at specified time points post transplantation (p.t.). **b** shows the change in absolute size of the 3–5 largest clones for each subject. **c** shows the oligoclonality index and the total number of unique integration sites for each subject over time

In Case 1 the degree of oligoclonal proliferation, measured by the oligoclonality index [16], peaked at day 59 and then plateaued, while the number of UIS slowly increased (Panel C). The absolute abundance of HTLV-1-infected clones was low compared with chronic infection acquired either from mother-to-child transmission or through sexual intercourse [16]. In both relative and absolute abundance clones present at day 61 were of lower abundance if redetected at day 186.

Case 2: At the first time point a large number of UIS (1031) were detected and the oligoclonality index was high; both these parameters subsequently decreased (Panel C). In this case the peak proviral load may be attributed to both an increase in the absolute number of integration sites (suggesting infectious spread) and vigorous expansion of a small number of clones.

Case 3: The oligoclonality index, reflecting clonal proliferation, peaked at day 48 and then declined over the next 3 months, with an increase in the absolute number of UIS consistent with infectious spread (Panel C).

### Estimated number of clones

The number of clones was estimated from the integration site high throughput sequencing data using a newly developed and published method, DivE. DivE compares multiple mathematical models fitted to rarefaction curves, which give the expected number of infected clones as a function of the number of infected cells. The estimated number of clones (median 1.3 × 10^5^) in these transplant recipients was substantially higher than those previously obtained in asymptomatic carriers (median 9.0 × 10^3^) [28], patients with HAM (median 2.8 × 10^4^) [29], and patients ATLL with (median 1.7 × 10^3^) [30].

## Discussion

For the majority of individuals, HTLV-1 is transmitted from mother-to-child via breast feeding and as such it is nearly impossible to conduct a study during acute infection. This report of an unfortunate clinical event has provided a rare opportunity to quantify the kinetics of acute HTLV-1 infection. We report that anti-HTLV-1 antibody responses were not delayed by immunosuppressive therapy: Anti-HTLV antibodies were detected in two cases at the first available time point (day 16) and in the third by day 39. This is similar to reports from recipients of HTLV-1-infected blood transfusions, in whom the seroconversion window has been estimated at 55 days [31], with 50 % having detectable HTLV-1 antibodies by day 40 [2], and contrasts with the recent report of delayed seroconversion in transplant recipients [4] .

We report that HTLV-1 spread early and rapidly in the three transplant recipients. In established HTLV-1 infection the proviral load varies more than 5 logs between individuals, and a high proviral load predicts and predates HTLV-1-associated disease. Proviral load remains stable within an individual over many years [32–34] but it has not been established how early this proviral load ‘set-point’ is reached. In the three recipients described here, who were immunosuppressed to different degrees, the ‘set-point’ proviral load was established by day 32–36 post-exposure after a rapid increase in proviral load with a median peak proviral doubling time of 1.4 days.

Early HTLV-1 spread is both ‘infectious’ and ‘mitotic’. We observed that the HTLV-1 2LTR DNA circles peaked with the peak proviral load, and both declined by the next testing time point. 2LTR DNA circles have not been previously reported in HTLV-1 infection and thus there are no data on the survival of such episomal viral DNA in HTLV-1 infection. The assumption that these 2LTR DNA circles are markers of recent infection is based upon this phenomenon in HIV [18]. Here, we provide evidence in HTLV-1 infection that 2LTR DNA circles are detected, and that they, or the cells in which they exist, are relatively short lived being much less frequent, as a proportion of infected cells, 2 weeks after the peak. The peak of 2 LTR DNA circles was observed after the introduction of oral antiretroviral therapy and there are two explanations for this: Firstly, whilst raltegravir has been demonstrated to prevent HTLV-1 spread from infected to uninfected cells in vitro [27], the therapy may not be active in vivo, even in primary infection due to reduced viral susceptibility. Secondly, early infectious spread may have already occurred, prior to the introduction of antiretroviral therapy, in a reservoir of inaccessible lymphoid tissue with later ‘spill over’ into the peripheral blood.

In each case, the peak oligoclonality index also coincided with the peak proviral load. The oligoclonality index reflects the degree of mitotic proliferation, which in case 2 at day 39 (Fig. 3, panel A) scored >0.8, in the range normally observed in ATLL. In each case, the oligoclonality index then decreased and stabilised in the range observed in chronic asymptomatic infection (0.3–0.55). The peak in the oligoclonality index also coincided with the peak frequency of 2LTR DNA circles. These results demonstrate both the expected early infectious spread and unexpected profound early mitotic proliferation which subsequently diminished.

It is unclear whether the therapeutic immune suppression during transplantation allowed rapid expansion of particular clones, which subsequently reduced in both absolute and relative abundance, or whether the period of antiretroviral therapy altered the balance between infectious spread and mitotic proliferation. Antiretroviral therapy was delivered by day 23 post-transplantation at therapeutically relevant doses but appeared unable to inhibit the early infectious spread of HTLV-1. In both in vitro [35, 36] and in vivo studies [25], zidovudine has previously been shown to effectively inhibit HTLV-1 infection. Similarly, raltegravir has also shown effectiveness in vitro [27]. The usefulness of these drugs in humans in early infection has never before been tested but these aforementioned studies provided the rational for their use here. It is possible that the effectiveness of these drugs in vitro and in animal models does not translate to humans or, as we believe more likely, that drug treatment was initiated after its potential therapeutic window.

In this setting, treatment with zidovudine and raltegravir did not control early infectious spread of HTLV-1. Since donor infection was diagnosed too late for post-exposure prophylaxis, antiretroviral therapy was initiated with the intention of limiting infectious spread during the most intense phase of immune suppression. The data suggest that the treatment started between day 16 and day 23 post-infection had no impact upon infectious spread and therefore once HTLV-1 proviral loads exceeded 1 % this treatment was discontinued. However, these results do not exclude a possible benefit of earlier post or peri-exposure prophylaxis.

Early onset and rapid progression of HAM, as previously reported [5, 6] was not observed. Further data are required on the long-term outcome of recipients of HTLV-1-infected organ transplants because in the context of an urgent life-saving transplant, such as liver or heart, the balance of risk and benefit may favour transplantation, even from a HTLV-1 infected donor.

## Conclusions

Following transplant-acquired infection: serological and molecular evidence of HTLV-1 can be detected as early as Day 16; the proviral set point is reached within 6 weeks and is the consequence of both mitotic and infectious spread; and there is no evidence that HTLV-1 enzyme inhibitors given from Day 16 of infection impact on the final proviral load.

## Methods

Relevant clinical details were abstracted from the medical records of each patient. In each case 8–11 sequential blood samples were obtained from day 16 until 21 months post-transplantation.

### Antibody detection

HTLV-1 antibodies were detected by Murex HTLV I + II (Diasorin Ltd, Dartford, UK) and confirmed by Western blot using Genelabs Diagnostics^®^ HTLV 2.4 assay (Genelabs, Redwood City, CA, USA) according to the manufacturer’s protocols.

### HTLV-1 proviral load

HTLV-1 proviral load was quantified by quantitative PCR (qPCR) as previously reported [32] with primers to the proviral *tax* and human *β*-*globin* gene, assuming one copy of *tax* [23] and two copies of *β*-*globin* per infected cell. Samples with unquantifiable provirus by qPCR (proviral load <0.01 % PBMCs) were amplified by nested PCR (nPCR) to confirm presence of provirus. The peak doubling time (T2) for proviral HTLV-1 was estimated from the sequential data as follows and a median result calculated:

$${\text{T2}} = \frac{\text{T}}{{{\text{Log }}\left( {{\text{V2}}/{\text{V1}}} \right)/{ \log }\,0. 5}},$$

where V1 and V2 are the proviral loads at the first and second time-points, respectively and T is the time between measurements.

### HTLV-1 long terminal repeat (LTR) sequencing

To identify the genotype of the HTLV-1 provirus, we amplified a 523 bp fragment between the LTR (5′-CTCGCATCTCTCCTTCACG-3′) and the *gag* gene (5′ CTGGTGGAAATCGTAACTGGA-3′). Cycling conditions: 98 °C for 3 min, 35 cycles 98 °C for 10 s, 64 °C for 20 s, 72 °C for 20 s followed by 72 °C for 10 min. The PCR products were electrophoresed on a 2 % agarose gel, inspected for length, and sequenced by Sanger sequencing.

### HTLV-1 2LTR DNA circles

PCR primers (Sigma, Poole, UK) for detection of unintegrated HTLV-1 2LTR DNA circles were designed by alignment with the AKT strain of the complete HTLV-1 genome (Accession Number J02029.1 available at http://www.ncbi.nlm.nih.gov/nuccore/J02029.1) whilst the NCBI Blast database program was used to confirm specificity. Primer sequences were as follows: outer-pX-forward: 5′-ATGAGCCCCAAATATCCCCCGGGG-3′, outer-pX-reverse: 5′-TCGATCTGTAACGGCGCAGAAC-3′, nested-pX-forward: 5′-AGCCACCGGGAACCACCCAT-3′, nested-gag-reverse: 5′-GACAAAGGCCCGGTCTCGACCT-3′. Classical PCR: sample DNA isolated from a known number of cells was amplified in 50 μl reaction volumes containing 0.1 µM of each outer primer, 200 μM dNTPs, 2 mM MgCl_2_, 1× green GoTaq reaction buffer (Promega, Southampton, UK) and 1.25u GoTaq DNA polymerase (Promega). Cycling conditions on an MJ Research PTC-225 (Bio-Rad, Hemel-Hempstead, UK) were: denaturation step 5 min at 94 °C, followed by 35 cycles of amplification consisting of 1 min at 95 °C, 30 s at 66 °C and 2 min at 72 °C, and a final elongation step of a further 5 min at 72 °C. For nested PCR, 1 µl of classical PCR product was transferred to 49 μl of reaction mix containing nested primers. Thermocycling conditions were: 94 °C for 5 min followed by 35 cycles consisting of 1 min at 95 °C, 30 s at 68 °C and 2 min at 72 °C, and a 5 min final elongation step at 72 °C. 10 µl of each reaction was separated on 2 % agarose gels containing 0.1 µg/ml ethidium bromide (Sigma) and visualised under UV light. LTR DNA circles were determined by serial dilution of purified sample DNA in water and amplification of quadruplicates at each dilution. LTR circle quantity was determined using Poisson’s distribution, where load = −log_n_ Fo^x^ dilution, and Fo is the number of negative tests/the number of tests. MT2 cells served as positive controls, DNA from HTLV-1 negative donor blood mononuclear cells and water as negative controls. 2LTR DNA circle frequency was calculated as the absolute number of 2LTR DNA circles per infected cell.

### Clonal abundance of Integration sites by high throughput sequencing (HTS)

A customised HTS protocol to map and accurately quantify proviral integration sites was used as previously described [16]. Fifty base-pair paired-end reads were acquired on an Illumina HiSeq 2000 analyser and the relative and absolute abundance of each clone deduced.

### Estimation of oligoclonality index (OCI)

As previously described, the OCI calculates the dispersion of a clonal population, describing the contribution of the largest clones to the total proviral load. An OCI close to 0 suggests a polyclonal population, where each clone occupies an equal share of the proviral load, whilst an OCI close to 1 suggests a dominant single clone [16].

### Diversity estimator (DivE)

DivE fits multiple mathematical models to nested subsamples of rarefaction curves, which depict the number of HTLV-1 infected clones against the number of HTLV-1 infected cells. Model performance is assessed by measuring the extent to which full data can be estimated from subsamples. Clonal diversity is estimated by extrapolating the best-performing models to a given population size (here the number of HTLV-1 infected cells in the circulation) [29]. A PBMC count of 3 × 10^9^/L was assumed in estimating the number of HTLV-1 infected cells.

## Ethics statement

The organs of the donor were offered for transplantation by the family of the donor, with written consent by the next-of-kin in accordance with the Human Tissues Act 2004 and the regulations of the Human Tissues Authority. In the United Kingdom this donation process and consent includes the testing of the donor for transplantation transmissible infections including HTLV-1.

Each recipient gave written informed consent for the organ transplantation which is documented in the medical records. In the UK verbal consent alone is sufficient to obtain blood samples for clinical purposes and this is not separately documented. Although some new investigation techniques were applied in the clinical investigation of this unusual event these were not performed as part of a research study and therefore in accordance with the guidance of the relevant authority, which in England is the Health Research Authority, application to the National Research Ethics Service is not required. Furthermore, each recipient gave their consent for the HTLV investigations and separately and specifically each gave consent, documented in their medical records, for the publication of their case and the results of their investigations.

## References

[1] group Iw. IARC monographs on the evaluation of carcinogenic risk to humans. World Health Organisation International Agency for Research on Cancer, vol 67. IARC Press. 1996. ISBN:9283212673

[2] Manns A, Murphy EL, Wilks R, Haynes G, Figueroa JP, Hanchard B (1991). Detection of early human T-cell lymphotropic virus type I antibody patterns during seroconversion among transfusion recipients. Blood.

[3] Emmanouilides CE, Territo M (1999). HTLV-I-associated myelopathy following allogeneic bone marrow transplantation. Bone Marrow Transpl.

[4] Glowacka I, Korn K, Potthoff SA, Lehmann U, Kreipe HH, Ivens K (2013). Delayed seroconversion and rapid onset of lymphoproliferative disease after transmission of human T-cell lymphotropic virus type 1 from a multiorgan donor. Clin Infect Dis Off Publ Infect Dis Soc Am.

[5] Gout O, Baulac M, Gessain A, Semah F, Saal F, Peries J (1990). Rapid development of myelopathy after HTLV-I infection acquired by transfusion during cardiac transplantation. N Engl J Med..

[6] Toro C, Rodes B, Poveda E, Soriano V (2003). Rapid development of subacute myelopathy in three organ transplant recipients after transmission of human T-cell lymphotropic virus type I from a single donor. Transplantation.

[7] Nakatsuji Y, Sugai F, Watanabe S, Kaido M, Koguchi K, Abe K (2000). HTLV-I-associated myelopathy manifested after renal transplantation. J Neurol Sci.

[8] Remesar MC, del Pozo AE, Pittis MG, Mangano AM, Sen L, Briones L (2000). Transmission of HTLV-I by kidney transplant. Transfusion..

[9] Younger DS (2015). HTLV-1-associated myelopathy/tropical spastic paraparesis and peripheral neuropathy following live-donor renal transplantation. Muscle Nerve.

[10] Nagamine Y, Hayashi T, Kato Y, Horiuchi Y, Tanahashi N (2015). Human T lymphotropic virus type-1-associated myelopathy manifesting shortly after living-donor renal transplantation. Intern Med.

[11] Kuroda Y, Takashima H, Yukitake M, Sakemi T (1992). Development of HTLV-I-associated myelopathy after blood transfusion in a patient with aplastic anemia and a recipient of a renal transplant. J Neurol Sci.

[12] Ramanan P, Deziel PJ, Norby SM, Yao JD, Garza I, Razonable RR (2014). Donor-transmitted HTLV-1-associated myelopathy in a kidney transplant recipient–case report and literature review. Am J Transpl Off J Am Soc Transpl Am Soc Transpl Surg.

[13] Cavrois M, Leclercq I, Gout O, Gessain A, Wain-Hobson S, Wattel E (1998). Persistent oligoclonal expansion of human T-cell leukemia virus type 1-infected circulating cells in patients with tropical spastic paraparesis/HTLV-1 associated myelopathy. Oncogene.

[14] Wattel E, Vartanian JP, Pannetier C, Wain-Hobson S (1995). Clonal expansion of human T-cell leukemia virus type I-infected cells in asymptomatic and symptomatic carriers without malignancy. J Virol.

[15] Meekings KN, Leipzig J, Bushman FD, Taylor GP, Bangham CR (2008). HTLV-1 integration into transcriptionally active genomic regions is associated with proviral expression and with HAM/TSP. PLoS Pathog.

[16] Gillet NA, Malani N, Melamed A, Gormley N, Carter R, Bentley D (2011). The host genomic environment of the provirus determines the abundance of HTLV-1-infected T-cell clones. Blood.

[17] Igakura T, Stinchcombe JC, Goon PK, Taylor GP, Weber JN, Griffiths GM (2003). Spread of HTLV-I between lymphocytes by virus-induced polarization of the cytoskeleton. Science.

[18] Sharkey M, Triques K, Kuritzkes DR, Stevenson M (2005). In vivo evidence for instability of episomal human immunodeficiency virus type 1 cDNA. J Virol.

[19] Sharkey ME, Teo I, Greenough T, Sharova N, Luzuriaga K, Sullivan JL (2000). Persistence of episomal HIV-1 infection intermediates in patients on highly active anti-retroviral therapy. Nat Med.

[20] Panther LA, Coombs RW, Aung SA, dela Rosa C, Gretch D, Corey L. Unintegrated HIV-1 circular 2-LTR proviral DNA as a marker of recently infected cells: relative effect of recombinant CD4, zidovudine, and saquinavir in vitro. J Med Virol. 1999;58(2):165–73. 10.1002/(sici)1096-9071(199906)58:2<165::aid-jmv11>3.0.co;2-1 10335865

[21] Cabral F, Arruda LB, de Araujo ML, Montanheiro P, Smid J, de Oliveira AC (2012). Detection of human T-cell lymphotropic virus type 1 in plasma samples. Virus Res.

[22] Demontis MA, Sadiq MT, Golz S, Taylor GP (2015). HTLV-1 viral RNA is detected rarely in plasma of HTLV-1 infected subjects. J Med Virol.

[23] Lairmore MD, Jason JM, Hartley TM, Khabbaz RF, De B, Evatt BL (1989). Absence of human T-cell lymphotropic virus type I coinfection in human immunodeficiency virus-infected hemophilic men. Blood.

[24] Hewitt PE, Davison K, Howell DR, Taylor GP (2013). Human T-lymphotropic virus lookback in NHS blood and transplant (England) reveals the efficacy of leukoreduction. Transfusion..

[25] Isono T, Ogawa K, Seto A (1990). Antiviral effect of zidovudine in the experimental model of adult T cell leukemia in rabbits. Leuk Res.

[26] Macchi B, Faraoni I, Zhang J, Grelli S, Favalli C, Mastino A (1997). AZT inhibits the transmission of human T cell leukaemia/lymphoma virus type I to adult peripheral blood mononuclear cells in vitro. J Gen Virol..

[27] Seegulam ME, Ratner L (2011). Integrase inhibitors effective against human T-cell leukemia virus type 1. Antimicrob Agents Chemother.

[28] Cook LB, Rowan AG, Melamed A, Taylor GP, Bangham CR (2012). HTLV-1-infected T cells contain a single integrated provirus in natural infection. Blood.

[29] Laydon DJ, Melamed A, Sim A, Gillet NA, Sim K, Darko S (2014). Quantification of HTLV-1 clonality and TCR diversity. PLoS Comput Biol.

[30] Cook LB, Melamed A, Niederer H, Valganon M, Laydon D, Foroni L (2014). The role of HTLV-1 clonality, proviral structure, and genomic integration site in adult T-cell leukemia/lymphoma. Blood.

[31] Schreiber GB, Busch MP, Kleinman SH, Korelitz JJ (1996). The risk of transfusion-transmitted viral infections. The Retrovirus Epidemiology Donor Study. N Engl J Med.

[32] Demontis MA, Hilburn S, Taylor GP (2013). Human T cell lymphotropic virus type 1 viral load variability and long-term trends in asymptomatic carriers and in patients with human T cell lymphotropic virus type 1-related diseases. AIDS Res Hum Retrovir.

[33] Iwanaga M, Watanabe T, Utsunomiya A, Okayama A, Uchimaru K, Koh KR (2010). Human T-cell leukemia virus type I (HTLV-1) proviral load and disease progression in asymptomatic HTLV-1 carriers: a nationwide prospective study in Japan. Blood.

[34] Taylor GP, Tosswill JH, Matutes E, Daenke S, Hall S, Bain BJ (1999). Prospective study of HTLV-I infection in an initially asymptomatic cohort. J Acquir Immune Defic Syndr.

[35] Macchi B, Balestrieri E, Mastino A (2003). Effects of nucleoside-based antiretroviral chemotherapy on human T cell leukaemia/lymphotropic virus type 1 (HTLV-1) infection in vitro. J Antimicrob Chemother.

[36] Matsushita S, Mitsuya H, Reitz MS, Broder S (1987). Pharmacological inhibition of in vitro infectivity of human T lymphotropic virus type I. J Clin Investig.

